# Clinical–EEG-Neuroimmunological Correlations in Depressive Female Adolescents with Non-Suicidal Self-Injuries

**DOI:** 10.1192/j.eurpsy.2022.827

**Published:** 2022-09-01

**Authors:** A. Iznak, E. Iznak, I. Oleichik, S. Zozulya

**Affiliations:** 1 Mental Health Research Centre, Laboratory Of Neurophysiology, Moscow, Russian Federation; 2 Mental Health Research Centre, Department Of Endogenous Mental Disorders And Affective Conditions, Moscow, Russian Federation; 3 Mental Health Research Centre, Laboratory Of Neuroimmunology, Moscow, Russian Federation

**Keywords:** Adolescents, non-suicidal self-injuries, quantitative EEG, neuroimmunology

## Abstract

**Introduction:**

Non-suicidal self-injurious behavior (NSSI) is rather widespread in adolescents, especially in females, and is an important risk factor for future suicide. The search for neurobiological markers of NSSI is therefore an actual medical and social problem.

**Objectives:**

The aim of the study was to determine the relationships between pre-treatment quantitative clinical, EEG, and neuroimmunological parameters in female depressive adolescents with NSSI.

**Methods:**

40 female depressive patients (all right-handed, 16–25 years old, mean 17.6±2.2 years old) were included in the study. Total HDRS-17 scores varied from 14 to 38 (mean 26.0±6.9). Multichannel eyes closed resting EEG was recorded with subsequent spectral power (SP) measurements in narrow frequency sub-bands. Enzymatic activities of leukocyte elastase (LE) and its antagonist α-1 proteinase inhibitor (α1-PI), as neuroinflammation markers, and the levels of autoantibodies to S100b protein (AAB-S100b) and to basic myelin protein (AAB-BMP), as neuroplasticity markers, were measured in the blood plasma. Spearman’s rank correlations were calculated between clinical, EEG, and neuroimmunological parameters.

**Results:**

HDRS-17 scores correlated positively (p<0.05) with beta2 (20-30 Hz) SP in the right anterior and mid-temporal leads. AAB-S100b values correlated positively with alpha2 (9-11 Hz) and alpha3 (11-13 Hz) SP in central-temporal-parietal-occipital leads, and correlated negatively with beta1 (13-20 Hz) and beta2 SP in the right anterior temporal lead.

**Conclusions:**

The structure of clinical–neurobiological correlations obtained indicates that processes of neuroinflammation in depressive adolescents with NSSI are relatively mild and/or compensated by anti-inflammatory mechanisms. The study supported by RBRF grant No.20-013-00129a.

**Disclosure:**

No significant relationships.

